# Low-Rank Tucker-2 Model for Multi-Subject fMRI Data Decomposition With Spatial Sparsity Constraint

**DOI:** 10.1109/TMI.2021.3122226

**Published:** 2022-03-02

**Authors:** Yue Han, Qiu-Hua Lin, Li-Dan Kuang, Xiao-Feng Gong, Fengyu Cong, Yu-Ping Wang, Vince D. Calhoun

**Affiliations:** School of Information and Communication Engineering, Dalian University of Technology, Dalian 116024, China; School of Information and Communication Engineering, Dalian University of Technology, Dalian 116024, China; School of Computer and Communication Engineering, Changsha University of Science and Technology, Changsha 410114, China; School of Information and Communication Engineering, Dalian University of Technology, Dalian 116024, China; School of Biomedical Engineering, Dalian University of Technology, Dalian 116024, China, and also with the Faculty of Information Technology, University of Jyväskylä, 40014 Jyväskylä, Finland; Department of Biomedical Engineering, Tulane University, New Orleans, LA 70118 USA; Tri-Institutional Center for Translational Research in Neuroimaging and Data Science (TReNDS), Georgia State University, Georgia Institute of Technology, Emory University, Atlanta, GA 30303 USA

**Keywords:** Tucker decomposition, multi-subject fMRI data, sparsity constraint, low-rank, core tensor

## Abstract

Tucker decomposition can provide an intuitive summary to understand brain function by decomposing multi-subject fMRI data into a core tensor and multiple factor matrices, and was mostly used to extract functional connectivity patterns across time/subjects using orthogonality constraints. However, these algorithms are unsuitable for extracting common spatial and temporal patterns across subjects due to distinct characteristics such as high-level noise. Motivated by a successful application of Tucker decomposition to image denoising and the intrinsic sparsity of spatial activations in fMRI, we propose a low-rank Tucker-2 model with spatial sparsity constraint to analyze multi-subject fMRI data. More precisely, we propose to impose a sparsity constraint on spatial maps by using an *ℓ*_*p*_ norm (0 < *p* ≤ 1), in addition to adding low-rank constraints on factor matrices via the Frobenius norm. We solve the constrained Tucker-2 model using alternating direction method of multipliers, and propose to update both sparsity and low-rank constrained spatial maps using half quadratic splitting. Moreover, we extract new spatial and temporal features in addition to subject-specific intensities from the core tensor, and use these features to classify multiple subjects. The results from both simulated and experimental fMRI data verify the improvement of the proposed method, compared with four related algorithms including robust Kronecker component analysis, Tucker decomposition with orthogonality constraints, canonical polyadic decomposition, and block term decomposition in extracting common spatial and temporal components across subjects. The spatial and temporal features extracted from the core tensor show promise for characterizing subjects within the same group of patients or healthy controls as well.

## I. INTRODUCTION

TENSOR decompositions have attracted increasing attention in blind source separation (BSS) of multi-subject functional magnetic resonance imaging (fMRI) data, since it can be readily represented by a three-way/mode tensor (voxel × time × subject). Canonical polyadic decomposition (CPD), Tucker decomposition (TKD), and block term decomposition (BTD) are commonly adopted forms for tensor decomposition. CPD decomposes a three-way tensor into a linear combination of a series of rank-one tensors, and each rank-one tensor is the outer product of three loading vectors [1], [2]. TKD decomposes a three-way tensor into a core tensor and three factor matrices. It can be seen as a complete form of CPD with polyadic but not necessarily canonical expansion in rank-1 terms [3], and CPD is a special case of TKD model with a diagonal core tensor [4], [5]. BTD decomposes a tensor into a sum of low multilinear rank tensors [6]–[8], thus is a complete form of TKD [6], [7] or a constrained TKD with a block diagonal core tensor [8]. Among the three forms, BTD was used to decompose 4-way fMRI tensor (e.g., 2D slices × time × subject) [9], while CPD and TKD were suitable to decompose three-way fMRI data [1], [2], [10], [11]. CPD has found its effectiveness in fMRI analyses by extracting spatial maps (SMs), time courses (TCs), and subject-specific intensities from multi-subject fMRI data [1], [2], which provide an intuitive decomposition for understanding brain function and mental disorders [12]–[16]. For TKD, the factor matrices reflect the affiliation of orthogonal bases along each mode [4], [13]. The model is named as Tucker-2 when one of the factor matrices (commonly the third mode) is an identity matrix, and Tucker-1 is a TKD model with two identity matrices [4]. As a complete form of CPD [5], Tucker-2 model can similarly obtain shared SMs and TCs from the two factor matrices and subject-specific intensities from the core tensor. But the biggest difference with CPD is that the core tensor of Tucker-2 model contains additional interactions between different components [4], [13] and highly compressed information for subjects [3], [17]. Some researchers studied the dynamic functional connectivity networks of fMRI using the core tensor of TKD [10], [11], [18], [19] and the core tensor was proven to carry features of original data in image processing [20] and analyses of electroencephalography(EEG) data [3], [17].

However, the decomposition of the TKD model without constraints is not unique; constraints should be imposed on factor matrices and/or the core tensor [4], [13], [17], [21]. Among other constraints, higher-order SVD (HOSVD) and higher-order orthogonal iteration (HOOI) are two TKD models with orthogonality constraints on the core tensor and factor matrices [13]. HOSVD decompose a tensor into orthogonal matrices and all-orthogonal core tensor, while HOOI iteratively updates an orthonormal basis of the dominant subspace [4]. HOSVD has been utilized to identify dynamic functional connectivity (FC) patterns of multi-subject fMRI data from a third-order [10] or a fourth-order tensor [18] (1D/2D connection × time × subject), or to extract connectivity networks from a fourth-order fMRI tensor computed from a single-subject fMRI data [19]. Al-Sharoa *et al.* proposed to extract FC patterns across time and subjects using HOOI based on the tensor formed by adjacency matrices [11]. HOOI was also applied to decompose four-way EEG data to obtain a core tensor and factor matrices in each mode [5], [21]. The core tensor can be sequentially used to train a standard classifier [5] or seen as the links among the components from different modes [21], and the factor matrices can be utilized to extract the components of frequency/time/channel/trial [21].

In addition to the orthogonality constraint, other constraints were also used in TKD-based image processing. Lu *et al.* proposed to recover clear images based on TKD, which obtains underlying tensor (clear image) with a low-tubal-rank using nuclear norm constraint, and a sparse residual tensor using sparsity constraint [22]. Besides the nuclear norm, Frobenius norm (*ℓ*_F_ norm) regularization can also improve low-rankness. Grussler *et al.* verified that the rank-constrained Frobenius norm can outperform the nuclear norm [23]. Traoré *et al.* proposed to enforce a sparsity constraint on the core tensor and low-rank (*ℓ*_F_ norm) constraints on factor matrices, and learnt reconstructed patch for inpainting images using the factor matrices and the core tensor [24]. The approach was also flexible to incorporate non-negativity and orthogonality constraints [24]. Bahri *et al.* proposed Kronecker component analysis (RKCA) method for image denoising. They justified that *ℓ*_F_ norm and nuclear norm penalties can yield the same optimal solutions of low-rank factor matrices. The model was established by imposing *ℓ*_F_ norm constraints on the separated dictionaries (Kronecker product of two factor matrices) and sparsity constraints on the core and residual tensors [25].

As mentioned above, existing TKD algorithms for fMRI analyses mostly utilize orthogonality constraints to extract FC patterns across time and subjects. These algorithms, however, are unsuitable for extracting common spatial and temporal patterns across subjects due to distinct characteristics such as high-level noise [26]. Motivated by the success of TKD-based image denoising algorithms such as RKCA, we propose to enforce fMRI-specific constraints on Tucker-2 model to analyze three-way multi-subject fMRI data (voxel × time × subject). First, the spatial activity of fMRI is proven to be sparse. Xu *et al.* investigated that only a small percentage (<10%) of the entire neuronal population can be fired in any brain region at any moment [27]. Lennie found that the number of activated neurons accounted for less than 1% in human cortex [28]. The sparsity characteristic of fMRI signal is more promising [29]. As such, we propose to impose a spatial sparsity constraint on the shared SMs, which to our best knowledge has not been used in TKD-based fMRI analyses. In addition, the factor matrices representing the shared SMs and TCs should be low-rank in order to extract principal spatial and temporal components, and the core tensor should be sparse to improve the uniqueness of the TKD solution [4]. Therefore, imposing low-rank constraints on factor matrices and adding sparsity constraint on the core tensor are essential in TKD of fMRI data.

In this study, we build a Tucker-2 model with both sparsity and low-rank constraints to represent the characteristics of three-way fMRI data (voxel × time × subject). More precisely, we propose to impose sparsity constraint on SMs via an *ℓ*_*p*_ norm regularization (0< *p* ≤1), which is often used in place of *ℓ*_0_ norm [30]. Besides, since previous researches have justified that low-rankness can be promoted by Frobenius norm [22]–[25], we impose low-rank (*ℓ*_F_ norm) constraints on factor matrices to detect principal SM and TC components shared by all subjects, and impose sparsity constraints via an *ℓ*_1_ norm regularization on the core tensor and the residual tensor to improve the uniqueness and performance. We estimate the core tensor and factor matrices by alternating direction method of multipliers (ADMM) and half quadratic splitting (HQS). In addition to the shared SMs and TCs estimates, we propose to explore novel information from the core tensor, and extract spatial and temporal features as well as individual intensities. These spatial and temporal features extracted from the core tensor are sequentially used to cluster multiple subjects into different groups. Simulated and experimental fMRI data are used to evaluate the proposed method.

To summarize, our contributions are as follows:

1. We propose a low-rank Tucker-2 model with a spatial sparsity constraint to decompose multi-subject fMRI data into shared SMs, shared TCs, and a core tensor including novel information in addition to subject-specific intensities.
2. We propose to enforce the sparsity constraint on SMs via an *ℓ*_*p*_ (0 < *p* ≤ 1) norm regularization, and adopt low-rank constraints on the two factor matrices via the Frobenius norm regularization and impose sparsity constraints on the core and residual tensors via the *ℓ*_1_ norm regularization. We solve the proposed Tucker-2 model using ADMM, and propose to update SMs with both sparsity and low-rank constraints using HQS.
3. We propose to explore the novel information embedded in the core tensor. In addition to the recovery of subject-specific intensities, we extract spatial and temporal features, which are then used to classify multiple subjects.

The rest of this paper is organized as follows. Section 2 describes the proposed method in detail. Section 3 presents the fMRI data used in our experiments and performance metrics adopted. Section 4 has the results from both simulated and experimental fMRI data. Section 5 includes the discussion of this study.

## II. METHODS

In this section, we first describe the proposed method, named as slcTKD (sparsity-low rank-constraints TKD). Afterwards, we introduce the update rules of factor matrices, core and residual tensors, as well as multipliers and penalty parameters. We then present a detailed implementation of the proposed slcTKD algorithm. Finally, we present the method of extracting the subject-specific intensities as well as spatial and temporal features.

### A. Notations

In the proposed method, tensors, matrices, and vectors are denoted by underlined bold capital letters (e.g., **X**), bold uppercase letters (e.g., **B**), and bold lowercase letters (e.g., **b**), respectively. Frontal (mode-3) slices of tensor **X** are defined as **X**_*k*_ = **X**(:, :, *k*), where “:” means that all samples are used. We denote the norm of tensor as ∥**X**∥ = ∑_*k*_∥**X**_*k*_∥, where “||·||” represents an *ℓ*_*p*_, *ℓ*_1_, or *ℓ*_F_ norm regularization. Table I includes the definitions of other notations used in this study.

### B. The Proposed slcTKD Model

Given a three-way (voxel × time × subject) multi-subject fMRI data $\underline{X}\in {\mathbb{R}}^{V\times T\times K}$, where *V* is the number of in-brain voxels, *T* is the number of time points, and *K* is the number of subjects. The Tucker-2 model of **X** is built as follows:

(1) $$\underline{X}=\underline{G}\times_{1}S\times_{2}B+\underline{E}$$

where $S\in {\mathbb{R}}^{V\times N}$ and $B\in {\mathbb{R}}^{T\times N}$ are two factor matrices corresponding to the shared SMs and TCs, and *N* is the number of components (i.e., the model order); $\underline{G}\in {\mathbb{R}}^{N\times N\times K}$ is the core tensor including subject-specific intensities, and spatial and temporal features; and $\underline{E}\in {\mathbb{R}}^{V\times T\times K}$ is the residual tensor.

Taking into account all sparsity and low-rank constraints imposed on each of factor matrices and tensors in the model (1), we propose slcTKD model for analyzing multi-subject fMRI data as follows:

(2) $$\underset{S,B,\underline{G},\underline{E}}{\min}\|\underline{X}-\underline{G}\times_{1}S\times_{2}B-\underline{E}{\|}_{F}^{2}+\|S{\|}_{\text{F}}^{2}+\|B{\|}_{\text{F}}^{2}\;\;+\delta \|S{\|}_{p}+\lambda \|\underline{G}{\|}_{1}+\gamma \|\underline{E}{\|}_{1}$$

where the sparsity constraint on **S** is imposed via the *ℓ*_*p*_ norm (0 < *p* ≤ 1); sparsity constraints on the core and residual tensors are imposed by the *ℓ*_1_ norm. Positive parameters *δ*, *λ*, and *γ* control the effects of the sparsity constraints, and the use of both *p* and *δ* makes the spatial sparsity level closer to that of actual SMs. The Frobenius norm of factor matrices **S** and **B** promote the low-rankness of shared SMs and TCs [25].

The problem in (2) is a convex optimization problem for each component individually. The work in [22], [31], and [32] exhibited the efficiency of ADMM in solving similar problems. We also take advantage of ADMM to update the factor matrices and tensors. By introducing a split variable **R** for the core tensor [31], the slcTKD model in (2) is rewritten as follows:

(3) $$\underset{S,B,\underline{G},\underline{E},\underline{R}}{\min}\|S{\|}_{\text{F}}^{2}+\|B{\|}_{\text{F}}^{2}+\delta \|S{\|}_{p}+\lambda \|\underline{G}{\|}_{1}+\gamma \|\underline{E}{\|}_{1}\;\;s\text{.}t.\;\underline{X}=\underline{R}\times_{1}S\times_{2}B+\underline{E}\;\;s\text{.}t.\;\underline{G}=\underline{R}$$

The mode-1 and mode-2 product of factor matrices with the core tensor can be expressed in terms of matrices multiplication:

(4) $$\underline{R}\times_{1}S\times_{2}B=\sum_{k}S{\underline{R}}_{k}B^{T}$$

As such, the augmented Lagrangian method [25], [31] derived from (3) is formulated as follows:

(5) $$L(S,B,\underline{G},\underline{E},\underline{R},\underline{U},\underline{W},\alpha ,\beta )\;=\|S{\|}_{\text{F}}^{2}+\|B{\|}_{\text{F}}^{2}+\delta \|S{\|}_{p}+\lambda \|\underline{G}{\|}_{1}\;\;\;\;\;+\gamma \|\underline{E}{\|}_{1}+\alpha \sum_{k}{\left\|{\underline{X}}_{k}-S{\underline{R}}_{k}{\underline{B}}^{T}-{\underline{E}}_{k}\right\|}_{\text{F}}^{2}+\beta \|\underline{G}-\underline{R}{\|}_{\text{F}}^{2}\;\;\;\;\;+\sum_{k}\langle {\underline{X}}_{k}-S{\underline{R}}_{k}{\underline{B}}^{T}-{\underline{E}}_{k},{\underline{U}}_{k}\rangle +\sum_{k}\langle {\underline{G}}_{k}-{\underline{R}}_{k},{\underline{W}}_{k}\rangle$$

where **U** and **W** are Lagrangian multipliers, and *α*, *β* are penalty parameters. By minimizing the augmented Lagrangian problem in (5), the factor matrices, tensors, multipliers, and penalty parameters are iteratively updated until convergence. We present update rules in detail in the following sub-sections.

### C. Update Rules of the Proposed slcTKD Model

#### 1) Updates of Two Factor Matrices B and S:

The update of factor matrix **B** can be derived from (5) by matrix algebra as follows:

(6) $$B=\frac{\sum_{k}{\left(\alpha \left({\underline{X}}_{k}-{\underline{E}}_{k}\right)+{\underline{U}}_{k}/2\right)}^{T}SR_{k}}{I+\alpha \sum_{k}{\underline{R}}_{k}^{T}S^{T}S{\underline{R}}_{k}}$$

Regarding the factor matrix **S** imposed both *ℓ*_F_ and *ℓ*_*p*_ constraints, it is difficult to be directly determined by matrix algebra. As such, we propose to utilize HQS algorithm to simplify the update of **S**. We add a variable **Y** into (5) and replace the *ℓ*_*p*_ norm regularization on **S** with a function of **Y**. The augmented Lagrangian approach for updating **S** and **Y** are in the following:

(7) $$L(S,B,\underline{G},\underline{E},\underline{R},\underline{U},\underline{W},Y,Q,\alpha ,\beta )\;\;\;\;\;\;\;=\|S{\|}_{\text{F}}^{2}+\delta \|S-Y{\|}_{\text{F}}^{2}+\xi \|Y{\|}_{p}\;\;\;\;\;\;\;\;\;+\alpha \sum_{k}{\left\|{\underline{X}}_{k}-S{\underline{R}}_{k}B^{T}-{\underline{E}}_{k}\right\|}_{\text{F}}^{2}\;\;\;\;\;\;\;\;\;+\sum_{k}\langle {\underline{X}}_{k}-S{\underline{R}}_{k}B^{T}-{\underline{E}}_{k},{\underline{U}}_{k}\rangle \;\;\;\;\;\;\;\;\;+\langle S-Y,Q\rangle +L_{1}(B,\underline{G},\underline{E},\underline{R},\underline{U},\underline{W},\alpha ,\beta )$$

where *ξ* > 0, **Q** is a Lagrangian multiplier, and *L*_1_ is a part of *L* in (5) without **S**. Then we update **S** by an algebraic calculation as follows:

(8) $$S=\frac{\sum_{k}\left(\alpha \left({\underline{X}}_{k}-{\underline{E}}_{k}\right)\;+\;{\underline{U}}_{k}/2\right)B{\underline{R}}_{k}^{T}\;+\;\delta Y\;+\;Q}{(\delta \;+\;1)I\;+\;\alpha \sum_{k}{\underline{R}}_{k}B^{T}B{\underline{R}}_{k}^{T}}$$

The variable **Y** can be obtained using Newton-Raphson method [33], which is carried out by several number of iterations. We calculate **Y** in each iteration as follows:

(9) $$Y_{d}=\xi p\cdot sgn(Y)\circ |Y{|}^{p-1}+\delta (S-Y)-Q$$

(10) $$Y_{dd}=\xi p(p-1)|Y{|}^{p-2}-\delta 1$$

(11) $$Y=Y-Y_{d}./Y_{dd}$$

where *p* is the value of *p* in *ℓ*_*p*_ norm, and **1** is an unit matrix which has the same size with **Y**, and the elements are all 1.

#### 2) Updates of Core Tensor G and Residual Tensor E:

We update the core tensor **G** using the soft-shrinkage method as follows:

(12) $$\underline{G}=\sum k\left(sgn\left({\underline{\tilde{G}}}_{k}\right)\circ max\left\{\left|{\underline{\tilde{G}}}_{k}\right|-\lambda /2\beta ,0\right\}\right)$$

where ${\underline{\tilde{G}}}_{k}={\underline{R}}_{k}-{\underline{W}}_{k}/2\beta$.

The split variable **R** in (5) satisfies discrete-time Sylvester equation [25] in the following form:

(13) $$\alpha S^{T}S{\underline{R}}_{k}B^{T}B+\beta {\underline{R}}_{k}+\beta {\underline{G}}_{k}+{\underline{W}}_{k}/2-{\underline{\tilde{R}}}_{k}=0$$

where ${\underline{\tilde{R}}}_{k}=S^{T}\left(\alpha {\underline{X}}_{k}-\alpha {\underline{E}}_{k}+{\underline{U}}_{k}/2\right)B$. In the proposed algorithm, equation (13) can be solved by solvers for discrete-time Sylvester equations [25], such as Hessenberg-Schur method [34] or Bartds-Stewart algorithm [35].

Based on (5), we can also update the residual tensor **E** by soft-shrinkage method via the following:

(14) $$\underline{E}=\sum k\left(sgn\left({\underline{\tilde{E}}}_{k}\right)\circ max\left\{\left|{\underline{\tilde{E}}}_{k}\right|-\gamma /2\alpha ,0\right\}\right)$$

where ${\underline{\tilde{E}}}_{k}={\underline{X}}_{k}-S{\underline{R}}_{k}B^{T}+{\underline{U}}_{k}/2\alpha$.

#### 3) Updates of Multipliers and Penalty Parameters:

In the proposed slcTKD algorithm, we update the multipliers and parameters after updates of all factor matrices and tensors in each iteration. Specifically, the multipliers **U**, **W**, and **Q** are updated in each iteration of ADMM method as follows:

(15) $$\underline{U}\leftarrow \underline{U}+\alpha \left(\underline{X}-\underline{R}\times_{1}S\times_{2}B-\underline{E}\right)$$

(16) $$\underline{W}\leftarrow \underline{W}+\beta (\underline{G}-\underline{R})$$

(17) Q←Q+δ(Y−S)

The penalty parameters *α* and *β* are updated using the parameter tuning method proposed in [25] as follows:

(18) α←ηα

(19) β←ηβ

where *η* > 1 is a fixed parameter representing the increase rates of *α* and *β*.

### D. Implementation of the Proposed slcTKD Algorithm

We use the result from HOSVD, which better matches Tucker-2 model than CPD-related algorithm such as CPD-GEVD [36], as the initialization values of **S**, **B**, and **G** to accelerate the convergence, and let **U** = **W** = **0**, **Q** = **0**, *α*_0_ = *K/*||**X**||_F_, and *β*_0_ = *K/*||**R**||_F_. We fix the parameters *δ*, *p*, *λ* and *γ* in the algorithm, and discuss the selection of four parameters in Section V. The implementation of slcTKD algorithm is summarized in Table II.

### E. Extraction of Subject-Specific Intensities and Spatial and Temporal Features From the Core Tensor

**Fig. 1.**
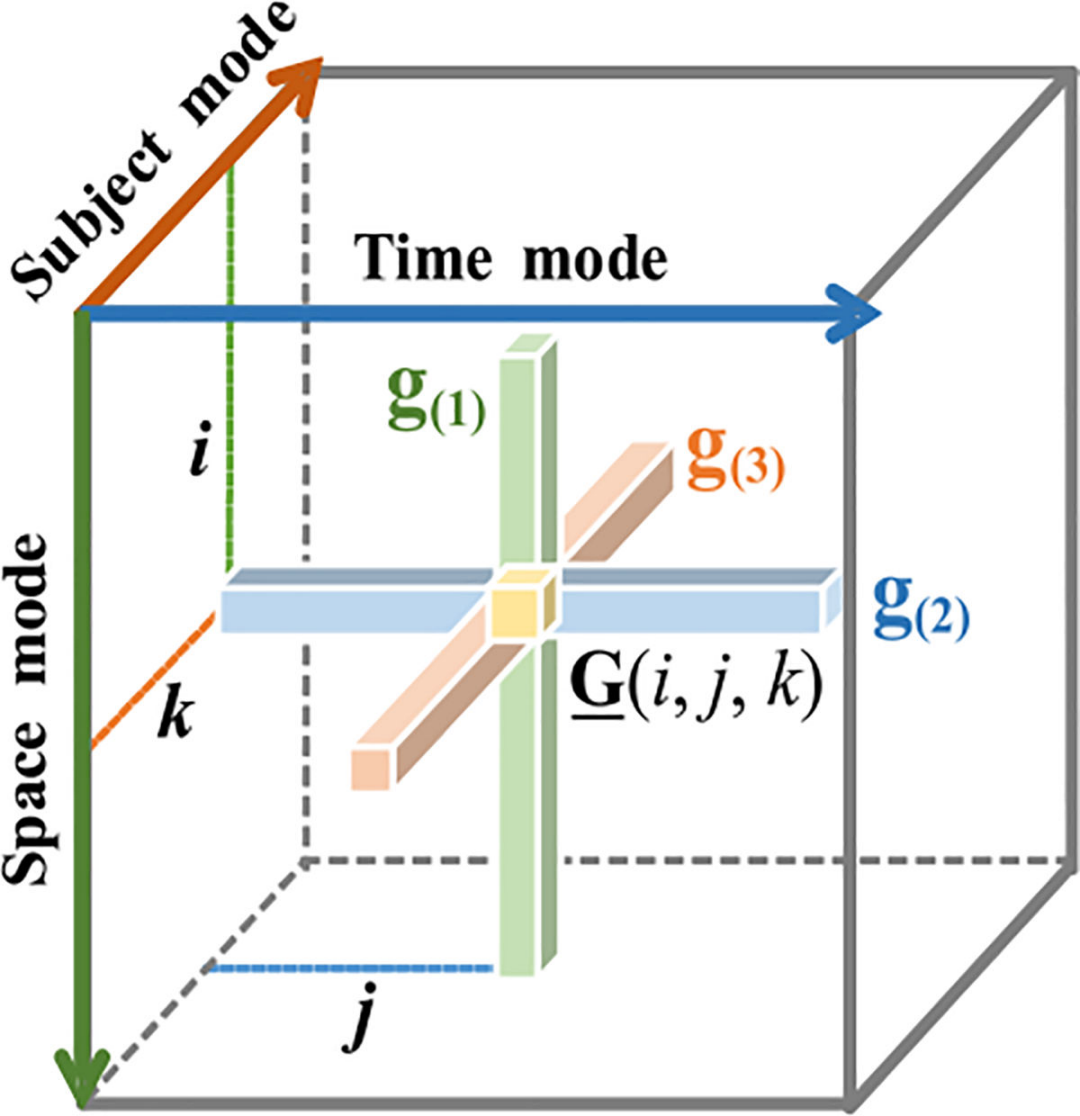
Fibers of the core tensor.

The slcTKD model provides a sparse, non-diagonal core tensor, which consists of spatial-temporal-subject information of the multi-subject fMRI data. When fixing the index of two modes, the three-way core tensor can be represented by fibers [4]. As Fig. 1 shows, we abbreviate the mode-1 (space mode), mode-2 (time mode) and mode-3 (subject mode) fibers as **g**_(1)_ = **G**(:, *j*, *k*), **g**_(2)_ = **G**(*i*, :, *k*), and **g**_(3)_ = **G**(*i*, *j*, :), respectively, where *i*, *j* = 1, 2, …, *N*, and *k* = 1, 2, …, *K*. Based on the fibers in different modes, we next extract the subject- specific intensities and the spatial and temporal features.

#### Subject-Specific Intensities:

We first extract subject-specific intensities by analyzing the core tensor. We rewrite the model in (1) by the form of frontal slices as follows:

(20) $${\underline{G}}_{k}=S^{†}\left({\underline{X}}_{k}-{\underline{E}}_{k}\right){\left(B^{T}\right)}^{†}$$

where ${\underline{G}}_{k}\in {\mathbb{R}}^{N\times N}$, ${\underline{X}}_{k}\in {\mathbb{R}}^{V\times T}$, ${\underline{E}}_{k}\in {\mathbb{R}}^{V\times T}$. We denote $\tilde{S}=S^{†}$, $\tilde{B}={\left(B^{T}\right)}^{†}$ then we can extract the *kth* component of mode-3 fibers of the core tensor as follows:

(21) $$g_{(3)k}={\tilde{s}}_{i}\left({\underline{X}}_{k}-{\underline{E}}_{k}\right){\tilde{b}}_{j}={\underline{G}}_{k}(i,j)$$

where **g**_(3)*k*_ = **g**_(3)_(*k*), ${\tilde{s}}_{i}=\tilde{S}(i,:)$, ${\tilde{b}}_{j}=\tilde{B}(:,j)$. We assume **s**_*i*_ = **S**(:, *i*) and obtain ${\tilde{s}}_{i}\cdot s_{i}=1$, where **s**_*i*_ is a spatial component in **S**, thus, ${\tilde{s}}_{i}$ is a spatial component related vector. Similarly, since **b**_*j*_ = **B**(:, *j*) and ${\tilde{b}}_{j}^{T}\cdot b_{j}=1$, ${\tilde{b}}_{j}$ is a temporal component related vector. Therefore, **g**_(3)*k*_ defined in (21) reflects the intensity of subject *k* under specific spatial (*i*) and temporal (*j*) components, and mode-3 fibers of the core tensor consist of subject-specific intensities:

(22) $$c=\left[g_{(3)1},g_{(3)2},\ldots ,g_{(3)K}\right]$$

#### Spatial and Temporal Features:

We exploit the relationship of core tensor **G** and spatial components **S** by rewriting (20) as follows:

(23) $${\underline{G}}_{k}=\tilde{S}\cdot {\underline{Z}}_{k}$$

where ${\underline{Z}}_{k}\in {\mathbb{R}}^{V\times N}$ is given by **Z**_*k*_ = (**X**_*k*_ − **E**_*k*_)(**B**^*T*^)^†^, representing temporal component related information for subject *k*. For a specific temporal component ${\underline{Z}}_{k}^{j}={\underline{Z}}_{k}(:,j)$, the spatial features can be extracted based on (23):

(24) $$g_{(1)i}={\tilde{s}}_{i}\cdot {\underline{Z}}_{k}^{j}$$

where **g**_(1)*i*_ = **g**_(1)_(*i*), *i* = 1, 2, …, *N*, so spatial features can be represented by mode-1 fibers of the core tensor. Furthermore, the spatial feature matrix $G_{S}\in {\mathbb{R}}^{N\times K}$ is obtained as follows:

(25) $$G_{S}(i,:)=\left[{\tilde{s}}_{i}\cdot {\underline{Z}}_{1}^{j},{\tilde{s}}_{i}\cdot {\underline{Z}}_{2}^{j},\ldots ,{\tilde{s}}_{i}\cdot {\underline{Z}}_{K}^{j}\right]$$

Since **G**_**S**_ consists of spatial features of all subjects, its straightforward application is to classify multiple subjects within the same group to emphasize the intra-group difference.

When extracting temporal features from the core tensor, we rewrite (20) to highlight temporal components **B** as follows:

(26) $${\underline{G}}_{k}={\underline{M}}_{k}{\left(B^{T}\right)}^{†}$$

where ${\underline{M}}_{k}\in {\mathbb{R}}^{N\times T}$ is represented by **M**_*k*_ = (**S**)^†^(**X**_*k*_ − **E**_*k*_) and includes spatial component related information for subject *k*. For a specific spatial component ${\underline{M}}_{k}^{i}={\underline{M}}_{k}(i,:)$, temporal features can be represented by mode-2 fibers of the core tensor:

(27) $$g_{(2)j}={\underline{M}}_{k}^{i}\cdot {\tilde{b}}_{j}$$

where **g**_(2)*j*_ = **g**_(2)_(*j*). Similar to the spatial features defined in (25), we have the temporal features of all subjects as follows:

(28) $$G_{B}(j,:)={\left[{\underline{M}}_{1}^{i}\cdot {\tilde{b}}_{j},{\underline{M}}_{2}^{i}\cdot {\tilde{b}}_{j},\ldots ,{\underline{M}}_{K}^{i}\cdot {\tilde{b}}_{j}\right]}^{T}$$

where $G_{B}\in {\mathbb{R}}^{N\times K}$ is the temporal feature matrix. This can also be utilized to cluster multiple subjects into different groups.

### III. EXPERIMENTAL METHODS

To evaluate the efficiency of the proposed slcTKD model, we carry out experiments on both simulated and experimental fMRI data. We compare the slcTKD algorithm with four related algorithms including RKCA [25], [31], TKD (HOOI) [37], CPD determined using nonlinear least squares [38], and the multilinear rank-(*L*, *L*, 1, 1) BTD proposed in [9] for decomposing 4-way multi-subject fMRI data $\underline{X}\in {\mathbb{R}}^{I_{x}\times I_{yz}\times T\times K}$(*I*_*x*_ × *I*_*y*_ × *I*_*z*_ denotes spatial dimensions). By formulating the matrix representation of each shared SM component into a low-rank matrix (of rank *L*), the multilinear rank-(*L*, *L*, 1, 1) BTD gives more flexibility in the spatial domain, compared to 3rd-order CPD.

We compute the multilinear rank-(*L*, *L*, 1, 1) BTD as follows: first, we combine the first and second dimensions of the 4-way fMRI data tensor to obtain a 3-way tensor and perform 3rd-order CPD for this tensor. Second, we matricize each column vector of the SM matrix, obtained as the first CPD factor matrix, into a matrix in an inverse manner to how the first and second dimensions of the 4-way fMRI data tensor are combined, and perform rank-*L* approximation to this matrix to obtain each rank-*L* component. Finally, we combine the two rank-*L* components to obtain the shared SM, and have the shared TC and the subject-specific intensity from the two rank-one modes. We determine *L* via testing all possible choices, and select *L* = 6 (from 2 ~ 30) for the simulated fMRI data and *L* = 10 (from 2 ~ 45) for the experimental fMRI data.

Each algorithm is run 10 times for each case. For CPD and BTD, subject-specific intensity is directly obtained. For slcTKD, RKCA, and TKD, we extract subject-specific intensities from the core tensor using (22). The initialization values of *λ* and *γ* of RKCA are the same as the proposed slcTKD method for a fair comparison. In the implementation of slcTKD, we use *p* = 0.3, *γ* = 0.6, *λ* = 0.4 for both simulated and experimental fMRI data. In addition, we select *δ* = 2.5 for simulated data and *δ* = 0.4 for experimental data. For other parameters, we set *η* = 1.3, the iteration number of Newton-Raphson method *iter_y* = 10, *ξ* = 0.4, and the stop criterion *iter*_max_ = 300, *ε*_min_ = 10^−7^, Δ*ε*_min_ = 10^−4^. Note *ε*^*iter*^ is calculated using *ε*^*iter*^ = ||**X** − **G** ×_1_ **S** ×_2_ **B** − **E**||_F_*/*||**X**||_F_ for slcTKD and RKCA, *ε*^*iter*^ = ||**X** − **G** ×_1_ **S** ×_2_ **B**||_F_*/*||**X**||_F_ for TKD and CPD, and $\varepsilon^{\text{iter}\;}={\left\|\underline{X}-\sum_{n=1}^{N}\left(S_{1_{n}}S_{2_{n}}^{T}\right)\circ b_{n}\circ c_{n}\right\|}_{\text{F}}/\|\underline{X}{\|}_{\text{F}}$ for BTD (**S**_1_ and **S**_2_ are two spatial factors of low-rank *L*). The same stop criterion is used for all the algorithms, i.e., *iter* ≥ *iter*_max_, *ε*^*iter*^ ≤ *ε*_min_, or Δ*ε*^*iter*^ ≤ Δ*ε*_min_.

### A. Simulated fMRI Data

**Fig. 2.**
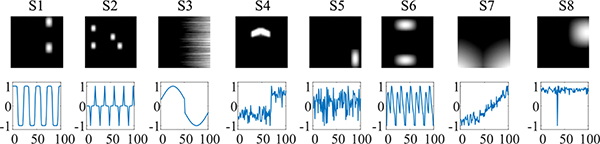
Eight simulated fMRI sources.

We generated 10 simulated fMRI subjects with each subject including eight fMRI-like sources, based on the benchmark data at http://mlsp.umbc.edu/resources.html [39], as shown in Fig. 2. 8 sources include task-related component (S1), transiently task-related components (S2 and S6), and artificial related components (S3, S4, S5, S7 and S8). Each spatial source contains 60 × 60 voxels with 100-point time courses. Each spatial map is reshaped into a one-dimensional vector (8 × 3600) and mixed with time courses (100 × 8). We obtain the real-valued simulated fMRI datasets (3600 × 100 × 10), each of which has randomly different activated SM voxels, i.e., randomly decrease of the activated SM voxels in the range of 10% [26]. In order to test the noise robustness of the slcTKD algorithm, we add Gaussian noise to the simulated fMRI data with different noise level. The signal-to-noise ratio (SNR) levels range from −10 dB to 10 dB with a 2.5 dB interval. The SNR is defined as 20log(*σ*_*s*_*/σ*_*n*_), where *σ*_*s*_ and *σ*_*n*_ are the temporal standard deviations of the source signal and Gaussian noise, respectively. Since components of interest cannot be well extracted by CPD and TKD when the model order is the same as the true number of components (*N* = 8), we choose a larger model order *N* = 20 for a fair comparison. Moreover, we test the effect of the model order on all algorithms by changing the model order in a range of 20 to 80 with an interval of 10.

### B. Experimental fMRI Data

The experimental fMRI dataset was collected from 10 subjects performing a finger-tapping motor task while receiving auditory instructions [16], [26]. All participants signed IRB-approved informed consent at the University of New Mexico. The experimental paradigm is a block design with alternating periods of 30 seconds on (finger tapping) and 30 seconds off (rest). The experiments were performed with a 3T Siemens TIM Trio system with a 12-channel receive coil. The fMRI experiment used a standard Siemens gradient-echo EPI sequence modified to store real and imaginary data separately, and we used the magnitude data in our experiment. The following parameters were used: field-of-view = 24 cm, slice thickness = 3.5 mm, slice gap = 1 mm, number of slices = 32, matrix size = 64 × 64, TE = 29 ms, TR = 2 s, flip angle = 70 degrees. The preprocessing of the data is carried out using Statistical Parametric Mapping (SPM) software package. The magnitude datasets were co-registered to compensate for movements in the fMRI time series images using INRIAlign. Images were then spatially normalized into the standard Montreal Neurological Institute space. Following spatial normalization, the data (originally acquired at 3.75 mm × 3.75 mm × 4.5 mm) were slightly sub-sampled to 3 mm × 3 mm × 3 mm, resulting in 53 × 63 × 46 voxels. Then, the images were spatially smoothed with a 10 mm × 10 mm × 10 mm full width at half-maximum Gaussian kernel.

After removing the voxels out of the brain and flattening the volume image data of 165 time points for each subject, we construct the three-way actual fMRI data of size 59610 × 165 × 10 (voxel × time × subject). The final model order *N* of each algorithm is set to 50, which is consistent with [16], [26].

### C. Performance Measures

For the simulated fMRI data, we choose the task-related component (S1) and two transiently task-related components (S2 and S6) as three components of interest. We calculate (1) the absolute Pearson correlation coefficients |*ρ*_*c*_| between the SM and TC estimates and the ground truths; (2) the mean of |*ρ*_*c*_| across 10 runs, denoted as $\left|{\bar{\rho }}_{c}\right|$, and the standard deviation.

For the experimental fMRI data, we select the task-related component and the default mode network (DMN) as two components of interest for evaluating the proposed method. For the task-related component, we utilize a group general linear model (GLM) map [16], [26] as the SM reference and the model TC as the TC reference. For DMN, we use the DMN component provided by Smith *et al.* [40] as the SM reference, and also use the model TC as the TC reference since DMN has the opposite trend to the task-related component. We calculate (1) the absolute Pearson correlation coefficient |*ρ*_*c*_| between the shared SM and TC estimates and their references; (2) the mean and standard deviation of |*ρ*_*c*_| values across 10 runs; (3) the total number of activated voxels as well as the voxels inside the spatial reference.

In addition, we test the performance of the subject-specific intensities as well as the spatial and temporal features. We first compare the subject-specific intensities among all five algorithms and calculate $\left|{\bar{\rho }}_{c}\right|$ values between each pair of the algorithms. Then, we test the performance of spatial and temporal features via k-means clustering [41]. The clustering results are verified using individual SM and TC references obtained by independent vector analysis [42].

## IV. RESULTS

### A. Simulated fMRI Data

**Fig. 3.**
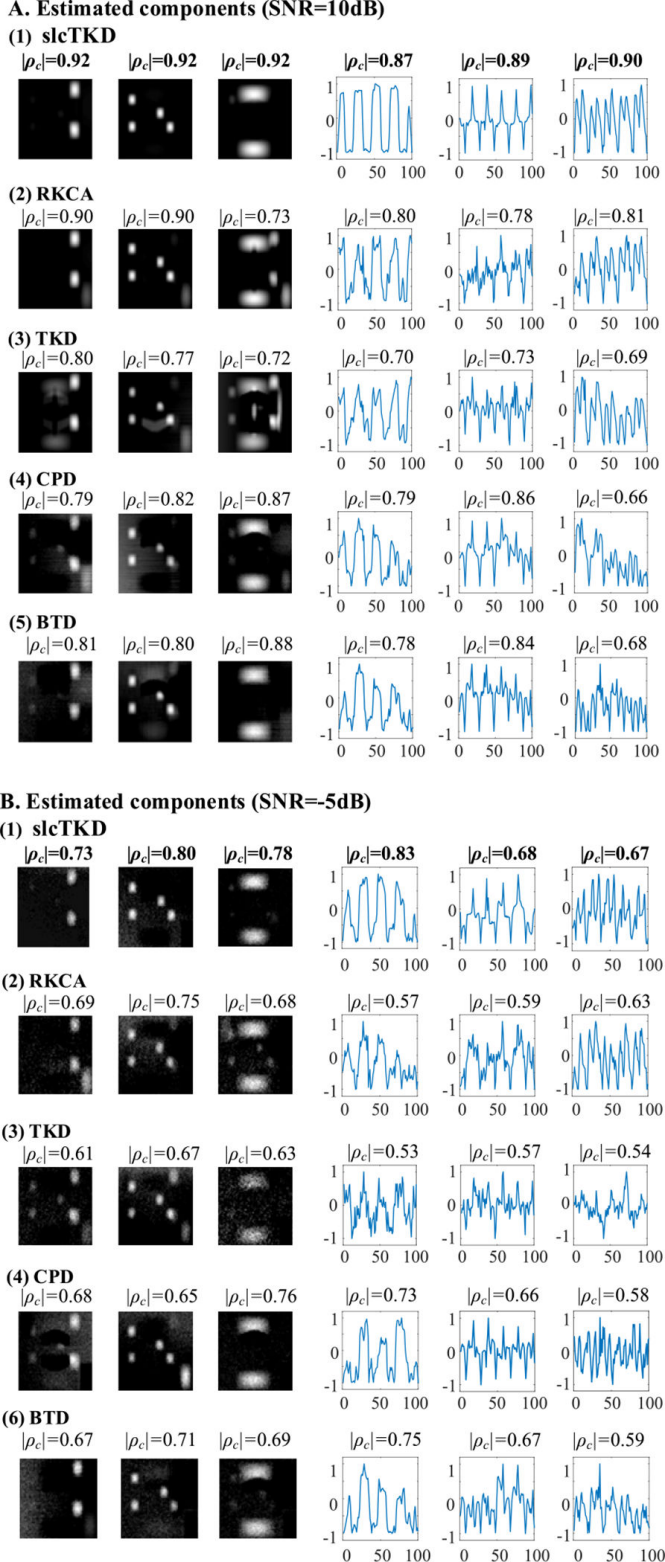
A comparison of shared SMs and TCs of S1, S2 and S6 estimated at (A) SNR=10dB and (B) SNR=−5dB by (1) slcTKD, (2) RKCA, (3) TKD, (4) CPD, and (5) BTD when *N* = 20. The maximum |*ρ*_*c*_| values are shown in bold.

We first test the noise effect on the proposed method. Fig. 3 shows the shared SMs and TCs estimated at SNR = 10dB and SNR = −5dB under the model order *N* = 20, since the differences of the algorithms are easy to be observed. For each algorithm, we select a run with the closest |*ρ*_*c*_| value to $\left|{\bar{\rho }}_{c}\right|$, i.e., the average of all runs, for both shared SMs and TCs. We detrend the TCs by baseline correction [43] and make normalization to the range of −1 to 1. From Fig. 3, we see that slcTKD yields the best performance among the five algorithms for both SMs and TCs; RKCA ranks second on average with much better SM estimates for S1 and S2, followed by BTD, CPD, and TKD.

**Fig. 4.**
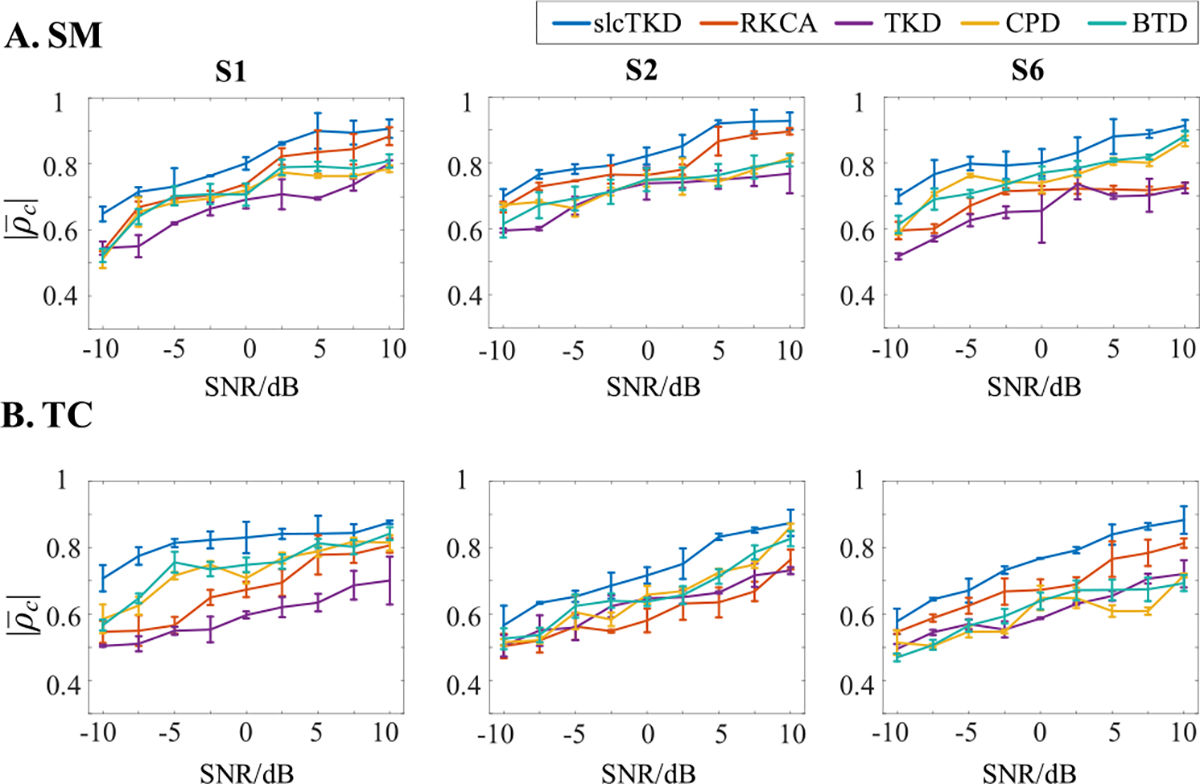
The comparison of $\left|{\bar{\rho }}_{c}\right|$ values and standard deviations for (A) shared SMs and (B) shared TCs of S1, S2 and S6 estimated by slcTKD, RKCA, TKD, CPD, and BTD at different noise levels when *N* = 20.

Fig. 4 shows the means and standard deviations of the |*ρ*_*c*_| values for the shared SMs and TCs across 10 runs at different noise levels when *N* = 20. We observe similar increasing trends with the increase of SNRs for the five approaches, but $\left|{\bar{\rho }}_{c}\right|$ values of the proposed slcTKD are the highest for both SMs and TCs from all three components. By contrast, the other four algorithms illustrate varying performance when estimating SMs and TCs for different components and under different SNRs. Taking S1 as example, RKCA performs much better than CPD when estimating SMs under higher SNRs, but it may become worse than CPD when estimating TCs under lower SNRs. The reason for this is that the low-rank constraint helps RKCA to obtain a better SM estimate for a sparse component (e.g., S1) at higher SNR levels, but cannot capture the SM characteristic at lower SNR levels, as a noisy SM is not sparse anymore. As a result, SM estimation degrades and so also do the TC estimates. CPD has no requirement on spatial sparsity; its degradation in SM estimation is slighter than RKCA, thus may provide better SM and TC estimates.

**Fig. 5.**
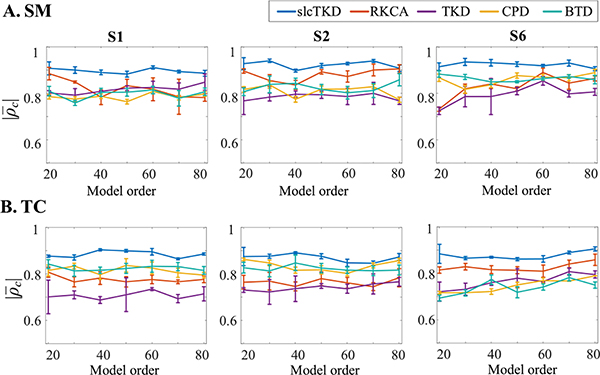
The comparison of $\left|{\bar{\rho }}_{c}\right|$ values and standard deviations for (A) shared SMs and (B) shared TCs of S1, S2 and S6 estimated by slcTKD, RKCA, TKD, CPD, and BTD in different model orders when SNR = 10dB.

Similarly, we test the model order effects on the proposed algorithm. Fig. 5 shows an exampling comparison of the means and standard deviations of |*ρ*_*c*_| values across 10 runs for SMs and TCs of the three components estimated by all five algorithms under different model orders for SNR = 10dB. We see that the proposed slcTKD algorithm generally achieves the highest $\left|{\bar{\rho }}_{c}\right|$ values and exhibits the least $\left|{\bar{\rho }}_{c}\right|$ changes with model order, while the other four algorithms cannot keep consistent performance when examining SMs and TCs for the three components.

### B. Experimental fMRI Data

**Fig. 6.**
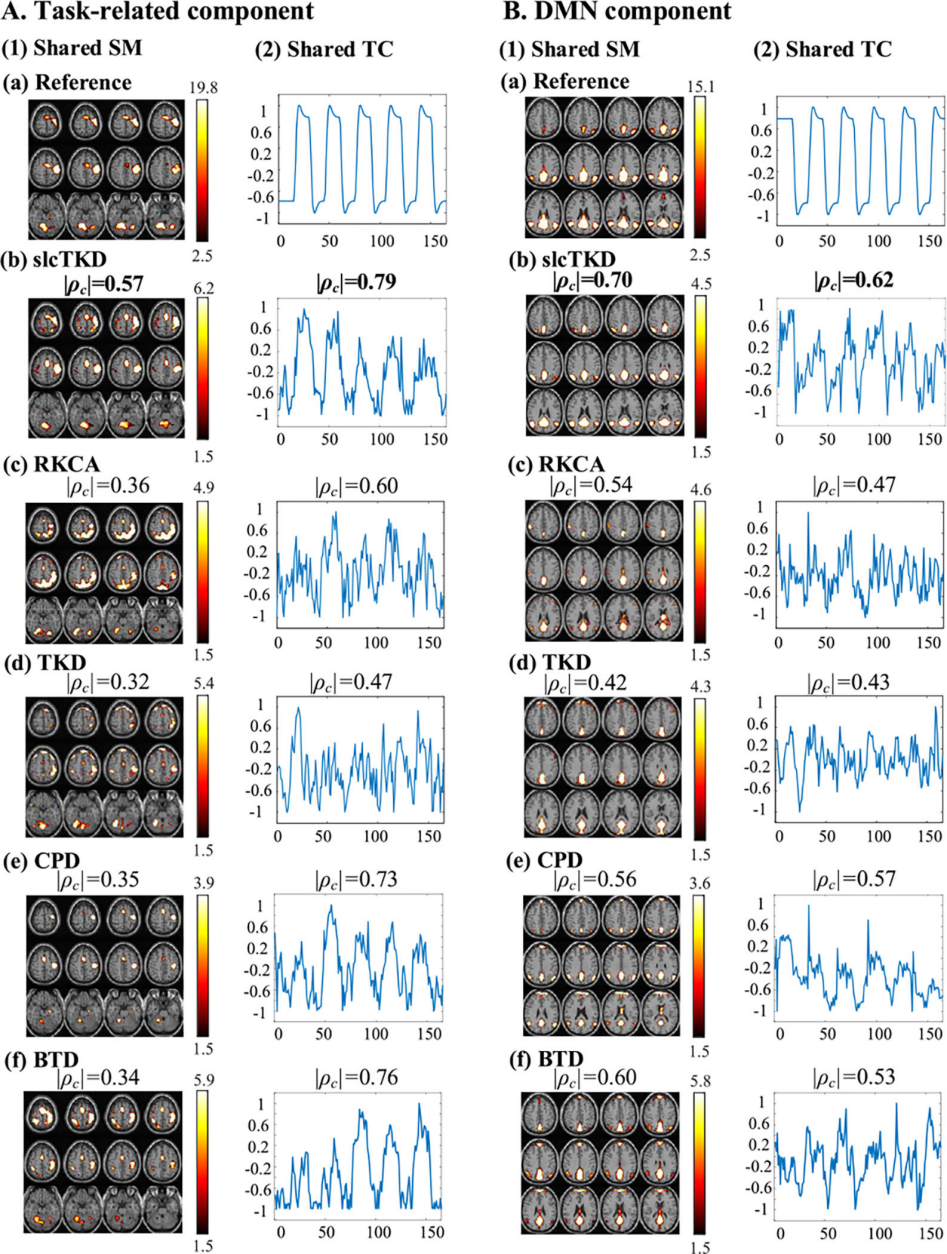
The comparison of (A) Task-related component and (B) DMN component estimated by slcTKD (b), RKCA (c), TKD (d), CPD (e), BTD (f). The estimates of shared SMs (1) and shared TCs (2) and their references (a) are shown. The maximum |*ρ*_*c*_| values are also shown in bold.

Fig. 6 shows shared SMs (|Z|>1.5) and shared TCs for the task-related and DMN components estimated by slcTKD, RKCA, TKD, CPD, and BTD, and their |*ρ*_*c*_| values with the spatial and temporal references. We show a single run with the closest |*ρ*_*c*_| value to the $\left|{\bar{\rho }}_{c}\right|$ value, i.e., the average of all runs, for both shared SMs and TCs. The TCs are baseline corrected [43] and normalized to the range of −1 to 1. The proposed slcTKD yields the best performance among the five algorithms when estimating both SMs and TCs for the two components, in terms of not only quantitative |*ρ*_*c*_| values but also qualitative observation (more similar to the SM and TC references than the other methods, though the estimated TCs of DMN are not so good as the task-related TC components in general). Unlike the simulated results, BTD and CPD are generally better than RKCA for both components and their SM and TC estimates, in terms of both quantitative |*ρ*_*c*_| values and qualitative observation. The results reflect that BTD and CPD are more suitable for modelling experimental fMRI data than RKCA without a spatial sparsity constraint, and the multilinear rank-(*L*, *L*, 1, 1) BTD improves SM estimation (e.g., DMN) due to low-rank formulating. This also verifies the lower SNR level of experimental fMRI data and the importance of sparsity constraints on SMs in Tucker-2 model. TKD obtains unsatisfactory results, suggesting its inadequate modeling for highly noisy experimental fMRI data.

Table III has results on the number of activated voxels for the two shared SMs estimated by the five algorithms displayed in Fig. 6. We compare the total number of activated voxel and voxel number inside the spatial reference. Although the proposed slcTKD does not detect the largest number of voxels in total, it does extract the largest number of voxels inside the spatial reference for both the task-related and DMN components. More precisely, slcTKD detects 31.1% ~ 135.6% more task-related voxels and 9.4% ~ 49.8% more DMN-related voxels, as compared to the other algorithms, providing larger and more contiguous sub-regions such as the supplementary motor area (SMA) of the task-related component and the posterior cingulate cortex (PCC) and inferior parietal lobule (IPL) for DMN.

**Fig. 7.**
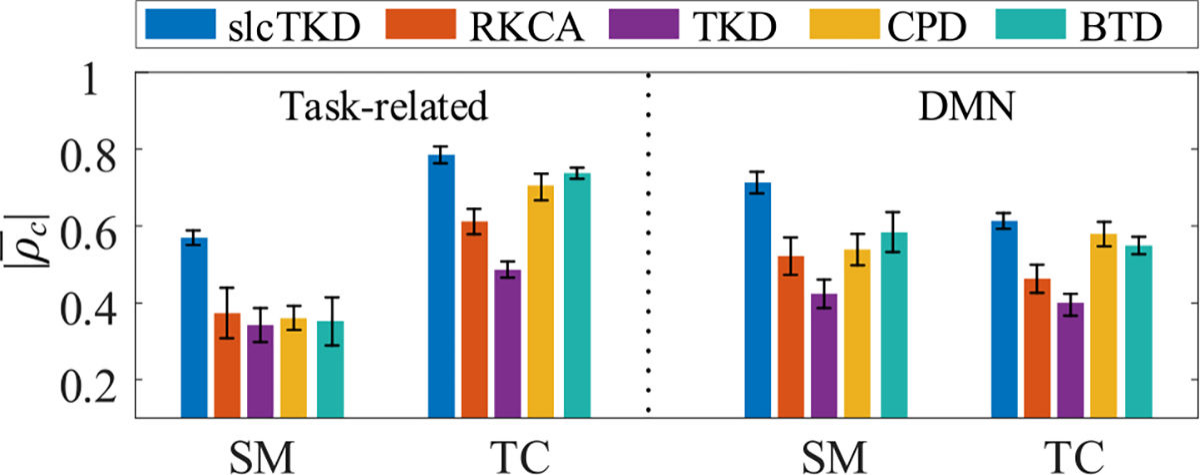
The comparison of |*ρ*_*c*_| values and standard deviations for shared SMs and shared TCs of task-related component and DMN component estimated by slcTKD, RKCA, TKD, CPD, and BTD.

Fig. 7 shows the mean and standard deviations of |*ρ*_*c*_| values for the shared SMs and TCs of the task-related and DMN components estimated by slcTKD, RKCA, TKD, CPD, and BTD, respectively, over all 10 runs for each algorithm. We can see that the proposed slcTKD algorithm achieves higher $\left|{\bar{\rho }}_{c}\right|$ values with a smaller standard deviation than the other four algorithms for both SMs and TCs of the task-related and DMN components, while TKD ranks the last. BTD and CPD performs better than RKCA for both SM and TC estimates of the two components in terms of both $\left|{\bar{\rho }}_{c}\right|$ values and standard deviations.

### C. Subject-Specific Intensities and Spatial and Temporal Features Extracted From the Core Tensor

**Fig. 8.**
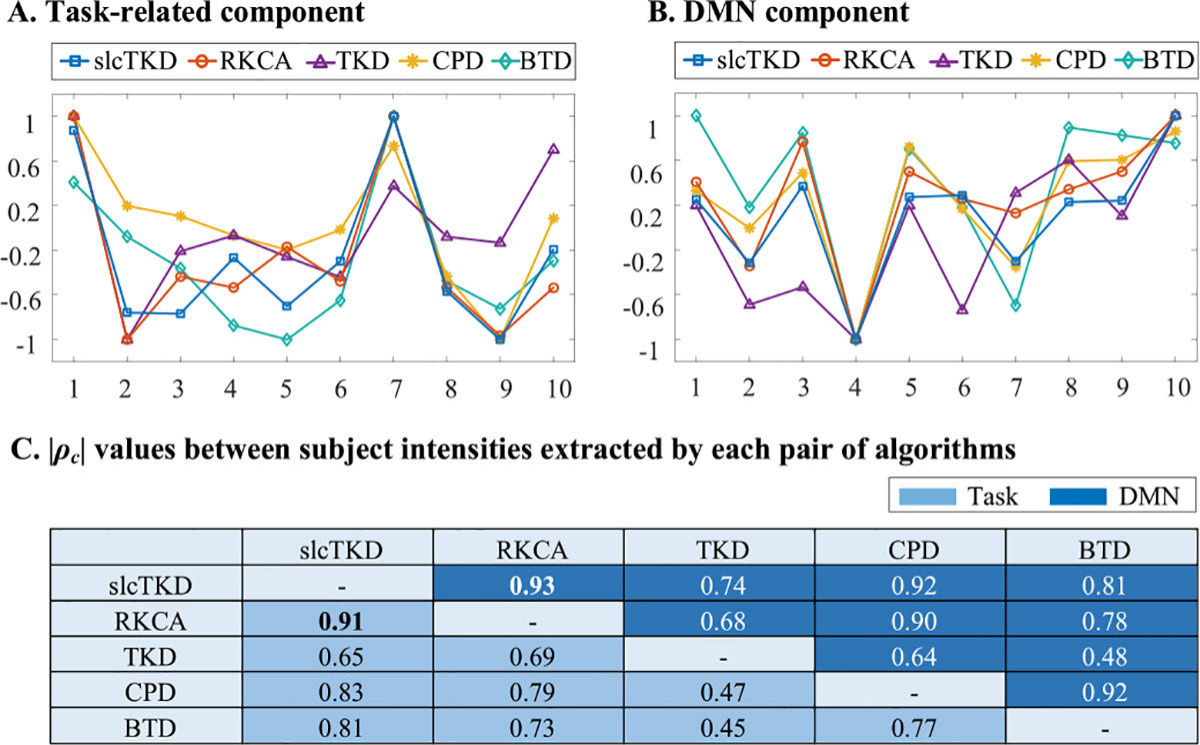
The comparison of subject-specific intensities extracted from the core tensor for (A) task-related component and (B) DMN component estimated by slcTKD, RKCA, TKD, CPD, and BTD, and |*ρ*_*c*_| values between subject-specific intensities extracted by each pair of algorithms (C).

Fig. 8 shows results of subject-specific intensities extracted from the core tensor for the task-related and DMN components, which are selected from the same run as that used in Fig. 6. They are normalized to the same range [−1, 1] for comparisons. These subject-specific intensities show mostly similar trends for both task-related component and DMN, especially for those from slcTKD, RKCA and CPD. Fig. 8C includes the |*ρ*_*c*_| values between the subject-specific intensities extracted by each pair of algorithms. It can be found that the |*ρ*_*c*_| values between slcTKD and RKCA are the largest (task-related: 0.91; DMN: 0.93) among 10 pairs of algorithms, since slcTKD and RKCA are based on a similar Tucker-2 model. The |*ρ*_*c*_| values between slcTKD and CPD rank the second largest, which are 0.83 and 0.92 for the task-related and DMN components, respectively. Note there can be large |*ρ*_*c*_| values between BTD and CPD (e.g., 0.92 for DMN) since we use CPD to compute the subject-specific intensities for BTD.

**Fig. 9.**
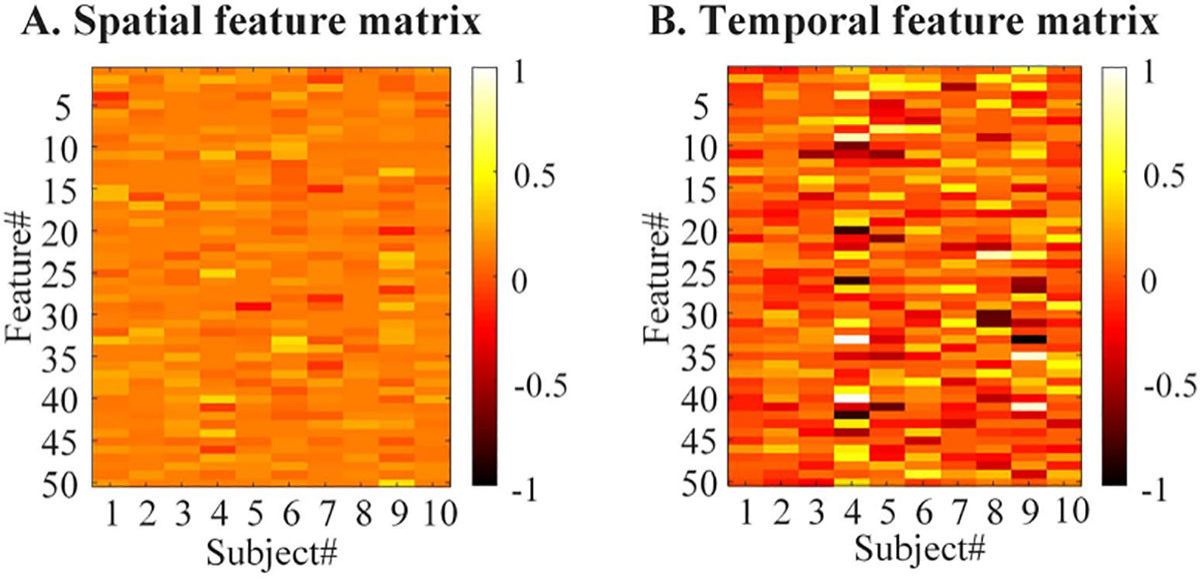
The spatial (A) and temporal (B) feature matrices extracted from the core tensor for the task-related component from 10 subjects.

**Fig. 10.**
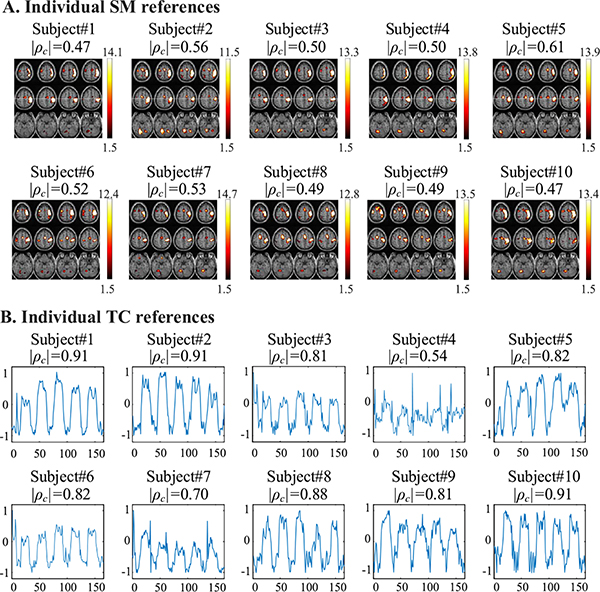
The individual SM (A) and TC (B) references for the task-related component. The |*ρ*_*c*_| values of these SM and TC references with the GLM reference and the model TC are also shown.

Fig. 9 shows spatial and temporal feature matrices extracted from the core tensor for the task-related component from the 10 subjects. Considering that intra-group spatial and temporal differences exist among a group of either patients or healthy subjects, due to individual anatomo-functional differences, individual cognitive strategies and specific subject variability (e.g., sex, age) [44]–[47], we utilize the spatial and temporal feature matrices to cluster multiple subjects into different subgroups. For comparisons, we generate individual SM and TC references by performing a group analysis on the experimental fMRI data using IVA-GL algorithm [42]. We run IVA-GL 10 times and extract the individual SM and TC references from the best run, as shown in Fig. 10. The |*ρ*_*c*_| values of these SM and TC references with the GLM reference and the model TC are also shown.

We basically cluster the 10 subjects into two groups by k-means clustering [41] based on the spatial and temporal feature matrices of the task-related component, respectively. When clustering using the spatial features, we obtain Group 1 = {subjects #1, #4, #7, #9, #10} and Group 2 = {subjects #2, #3, #5, #6, #8}. We can see that subject #3 and subject #4 are not in the same group though the |*ρ*_*c*_| values are the same (0.50), as shown in Fig. 10A. In fact, we can observe different task-related activations for subject #3 and subject #4 from Fig. 10A. This difference agrees with the inter-subject spatial variability reported in [44] and [45] and can be well captured by the spatial features involved in the core tensor.

When clustering subjects using the temporal features, we have Group 1 = {subjects #4} and Group 2 = {subjects #1, #2, #3, #5, #6, #7, #8, #9, #10}. This clustering is also reasonable by examining the individual TC references shown in Fig. 10B. The TC of subject #4 is significantly different from the others in that its |*ρ*_*c*_| value (0.54) is largely lower than those for the others (0.70 ~ 0.91). This result also agrees with the inter-subject temporal variability published in [46] and [47].

**Fig. 11.**
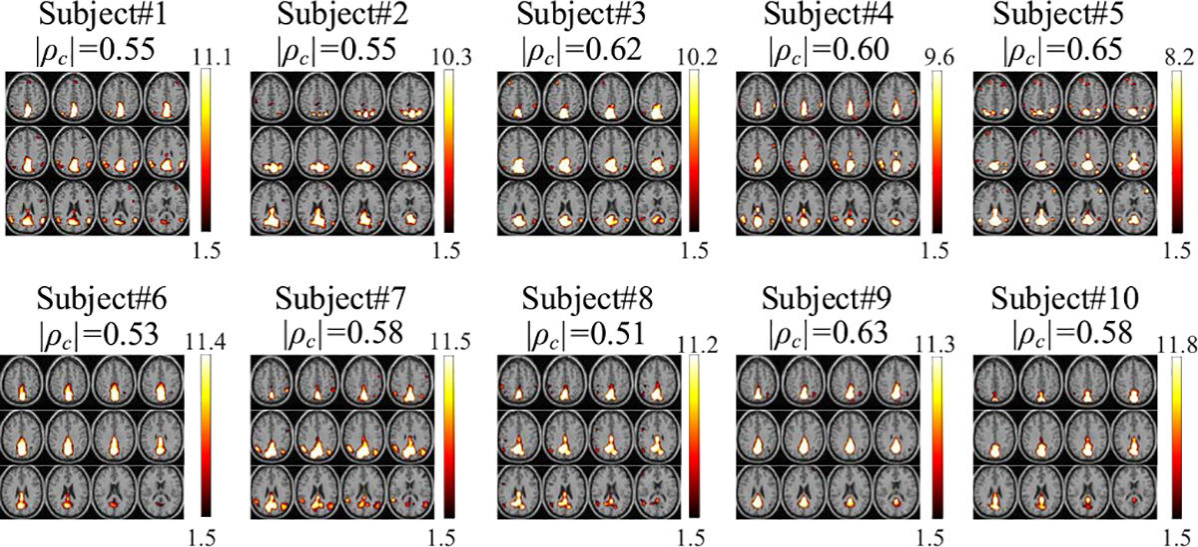
The individual SM references for the DMN component.

We also perform similar clustering based on the spatial features of DMN extracted from the core tensor. Fig. 11 shows the individual SM references separated from the experimental multi-subject fMRI data by the IVA-GL algorithm [42]. As a result, we obtain Group 1 = {subjects #1, #4, #5, #7} and Group 2 = {subjects #2, #3, #6, #8, #9, #10}. We see that subjects #1 & #2 and subjects #7 & #10 are not classified into the same group based on spatial features though their |*ρ*_*c*_| values are the same (0.55 and 0.58, as shown in Fig. 11). The spatial features extracted from the core tensor can capture spatial differences across subjects, e.g., IPL activations in Group 1.

## V. DISCUSSION

This study proposes a constrained Tucker-2 model for analyzing multi-subject fMRI data. We impose the sparsity constraint on SMs to incorporate the intrinsic characteristics of fMRI data, in addition to the low-rank constraint on factor matrices and the sparsity constraint on the core tensor. Compared with the mostly used CPD, the proposed method can provide not only shared SMs, shared TCs, and subject-specific intensities, but also the novel spatial and temporal features capturing spatial and temporal intra-group differences. The results from both simulated and experimental fMRI data verify the advantage of the proposed slcTKD algorithm over BTD, CPD, RKCA, and TKD.

While we can extract shared SMs, shared TCs, and subject-specific intensities agreeing with previous results of CPD-related methods [2], [12], [16], [26], we especially treasure the rich and unique spatial-temporal-subject information involved in the core tensor, which is missing in CPD of multi-subject fMRI data. Previously, researchers have utilized Tucker core tensor to build connectivity maps based on correlation matrices of multi-subject fMRI data, but they rarely used the spatially and temporally compressed information embedded in the core tensor for individual subjects. We extract novel spatial and temporal features apart from the subject-specific intensities from the core tensor, and apply these spatial and temporal features to make an intra-group clustering. The results show that these features can identify detailed differences in spatial activations or temporal responses across subjects, which extensively existed [44]–[47] but is hard to be evaluated by correlations with the references. Therefore, the proposed method show promise for providing new and reasonable spatial/temporal features to tell intra-group difference, as well as the shared SMs and TCs to provide common spatial/temporal features within a group.

### A. Effects of Constraints

The sparsity and low-rank constraints on the spatial factors are both essential for the proposed model to successfully extract principal spatial components, and the spatial sparsity constraint is vital for matching the spatial activation characteristics from fMRI data. This can be found by comparing the proposed slcTKD algorithm and RKCA using only a low-rank constraint on the spatial factors. RKCA exhibited appealing properties in image processing such as background subtraction and image denoising [25], [31] but degraded in the fMRI analysis. The main reason is that spatial maps of fMRI are much sparser than optical images. Therefore, the sparsity constraint contributes more to yield better SM estimates in the proposed algorithm. As a result, the proposed slcTKD algorithm removes a larger number of noisy voxels and recovers a larger number of voxels inside the spatial reference with more contiguous activations, as shown in Fig. 6. The high-quality SM estimates would further improve the estimates of spatial-temporal link and the shared TCs, which can be seen from their relationship in (20).

**Fig. 12.**
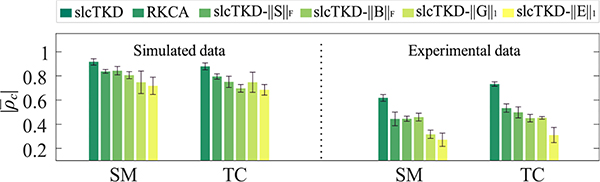
Comparison of the $\left|{\bar{\rho }}_{c}\right|$ values and standard deviations of shared SM and TC estimates over 10 runs of slcTKD-||S||_F_, slcTKD-||B||_F_, slcTKD-||G||_1_, and slcTKD-||E||_1_, which remove $\|S{\|}_{\text{F}}^{2}$, $\|B{\|}_{F}^{2}$, ||**G**||_1_, and ||**E**||_1_ from slcTKD, respectively. The results of slcTKD and RKCA are also shown for comparison.

**Fig. 13.**
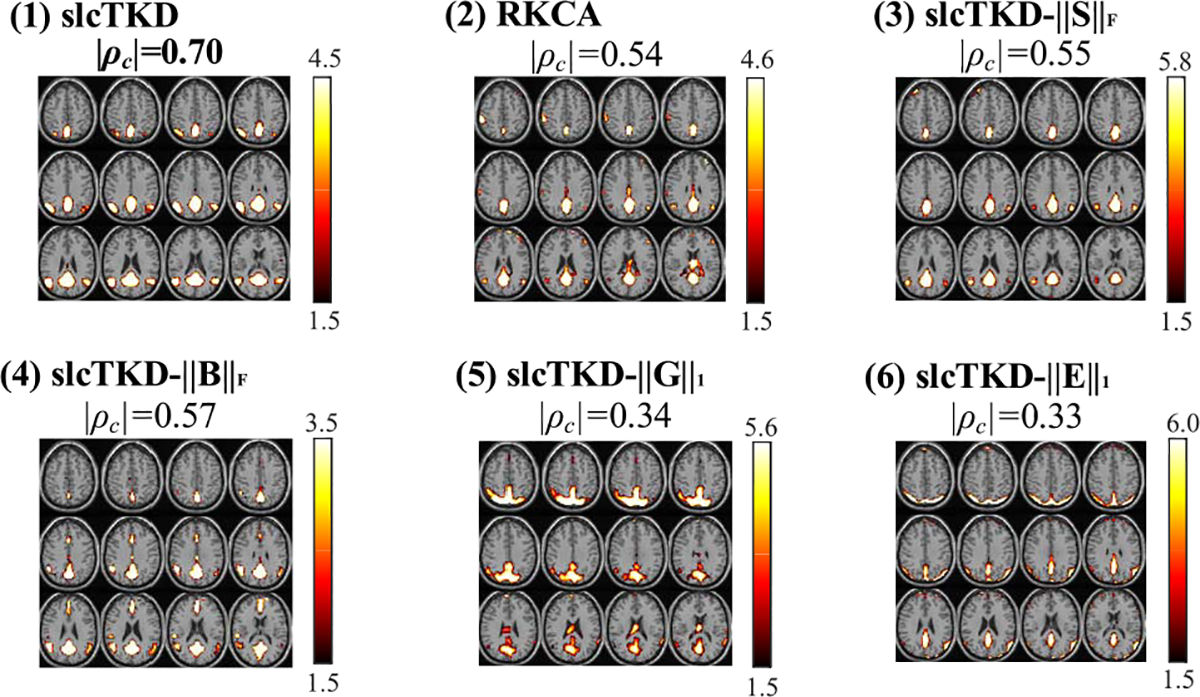
Comparison of the shared SMs of DMN estimated by (1) slcTKD, (2) RKCA, (3) slcTKD-||S||_F_, (4) slcTKD-||B||_F_, (5) slcTKD-||G||_1_, (6) slcTKD-||E||_1_. Themaximum |*ρ*_c_| value is shown in bold.

In addition, the low-rankness and sparsity constraints imposed on the factor matrices and the core and residual tensors are also necessary for the proposed slcTKD model to get good performance. The low-rank constraints $\|S{\|}_{\text{F}}^{2}$ and $\|B{\|}_{\text{F}}^{2}$ ensure the extraction of principal spatial and temporal components shared by all subjects, whereas sparsity constraints ||**G**||_1_ and ||**E**||_1_ improve the uniqueness and performance of the proposed method. To test effects of each constraint on the proposed algorithm, we respectively remove constraints $\|S{\|}_{\text{F}}^{2}$, $\|B{\|}_{\text{F}}^{2}$, ||**G**||_1_ and ||**E**||_1_ from slcTKD to have four algorithms, named as slcTKD-||S||_F_, slcTKD-||B||_F_, slcTKD-||G||_1_, and slcTKD-||E||_1_. We run each algorithm 10 times. Fig. 12 shows the mean and standard deviations of |*ρ*_*c*_| values for the shared SMs and TCs estimated by each algorithm, with comparison to the results of slcTKD and RKCA. Fig. 13 illustrates examples of shared SMs for DMN selected from a run with the closest |*ρ*_*c*_| value to $\left|{\bar{\rho }}_{c}\right|$ shown in Fig. 12 for the experimental fMRI data. We see that slcTKD has much higher $\left|{\bar{\rho }}_{c}\right|$ values than the other five methods. The two methods removing the sparsity constraints ||**G**||_1_ and ||**E**||_1_ from slcTKD result in lower $\left|{\bar{\rho }}_{c}\right|$ and noisier SMs than those removing the low-rankness constraints $\|S{\|}_{\text{F}}^{2}$ and $\|B{\|}_{\text{F}}^{2}$, while RKCA removing the sparsity constraint ||**S**||_*p*_ from slcTKD obtains a noisier SM than slcTKD-||S||_F_ and slcTKD-||B||_F_. These results verify that each constraint is necessary to the proposed method, and the sparsity constraints are especially essential for decomposing noisy multi-subject fMRI data using a Tucker-2 model.

### B. Effects of Parameters

The proposed method is mainly affected by parameters from the model constraints (sparsity and low-rankness) and the estimation algorithms (ADMM and HQS).

#### 1) Sparsity Constraint Parameters:

**Fig. 14.**
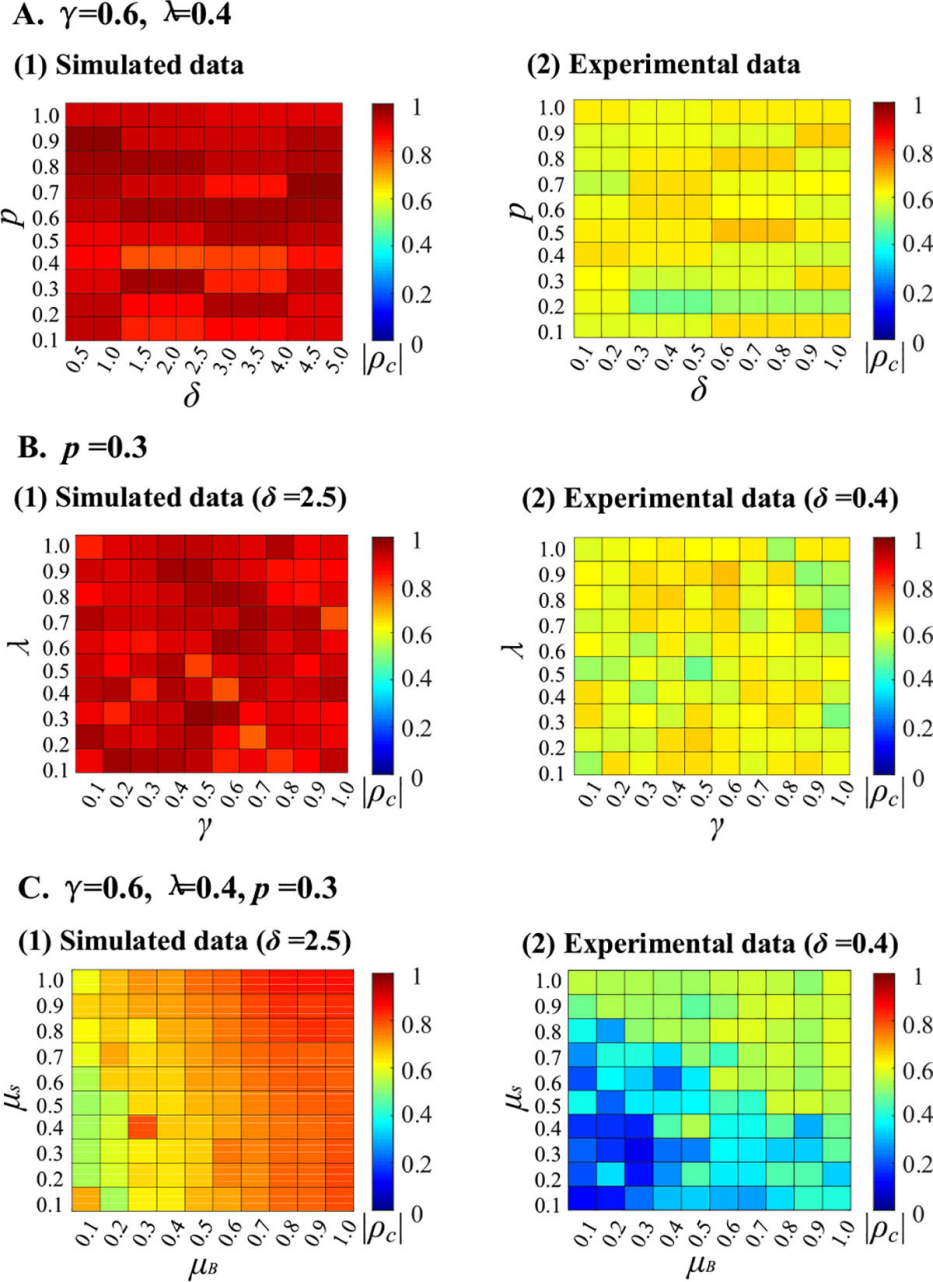
Parameters *δ* and p(A), *λ* and *γ* (B), and *μ*_S_ and *μ*_B_ (C) effects on the slcTKD model for simulated (1) and experimental (2) fMRI data.

The sparsity constraint parameters include *δ*, *p*, *λ* and *γ*, where *δ* and *p* are related to the spatial sparsity, and *λ* and *γ* are related to the sparsity of the core and residual tensors. Thus, we classify the four parameters into two groups, i.e., group 1 = {*δ* and *p*} and group 2 = {*λ* and *γ*}. We test the parameter effects in one group by fixing the parameters in the other by using the same initial values of **B**, **S** and **G**. We first test the parameter effects in group 1. The parameter *p* is changed from 0.1 to 1 at an interval of 0.1 for both simulated and experimental data. Considering the sparsity difference between two datasets, we change *δ* from 0.5 to 5 at an interval of 0.5 for the simulated data, while from 0.1 to 1 at an interval of 0.1 for the experimental data. Fig. 14A shows the |*ρ*_*c*_| values of the task-related SMs with *λ* = 0.4 and *γ* = 0.6, which are utilized in the experiments. We can see that the |*ρ*_*c*_| values are generally large and effects are slight. When testing the parameter effects in group 2, we change both *λ* and *γ* from 0.1 to 1 at an interval of 0.1 for both simulated and experimental data. The results in Fig. 14B shows that the parameters *λ* and *γ* have larger effects on the experimental data than simulated data, which is reasonable because of the different sparsity level of SMs from different subjects.

In summary, we use the same values of *p*, *λ* and *γ* for the simulated and experimental data but different value of *δ* to match their spatial sparsity difference. The sparser the spatial activations of the dataset are, the larger the *δ* value is. A larger *δ* is used for simulated fMRI data than for experimental fMRI data ( *δ* = 2.5 vs. *δ* = 0.4) because the simulated data is much sparser than experimental fMRI data in spatial activations. Note the same sparsity parameters are effective to extract all the sources for a specific (simulated/experimental) fMRI dataset [48]. Thus, our choices (*p* = 0.3, *δ* = 0.4, *γ* = 0.6, *λ* = 0.4) can be a good start to fine tune the four parameters for analyzing other experimental fMRI data.

#### 2) Low-Rank Constraint Parameters:

The shared SMs and TCs are extracted in pairs by the proposed method to represent associated spatial and temporal brain activity. Thus, we select the same low-rankness parameters for the two factor matrices **S** and **B**. We verify the effectiveness of doing this by rewriting the two terms of low-rank constraints in (2) as $\mu_{S}\|S{\|}_{\text{F}}^{2}$ and $\mu_{B}\|B{\|}_{\text{F}}^{2}$, and by changing *μ*_*S*_ and *μ*_*B*_ from 0.1 to 1 at an interval of 0.1 to test their effects. The |*ρ*_*c*_| values are shown in Fig. 14C. For both simulated and experimental fMRI data, the larger the parameters *μ*_*S*_ and *μ*_*B*_ are, the larger the |*ρ*_*c*_| values are. Therefore, *μ*_*S*_ = *μ*_*B*_ = 1 is a good and simple choice.

#### 3) ADMM and HQS Parameters:

The parameters from estimation algorithms consist of *η* for ADMM in (18) and (19), and *ξ* for HQS in (7). A previous *η* setting for RKCA [25], [31] (*η* = 1.3) is recommended. Comparing (7) with (2), the role of *ξ* is similar to that of *δ*, but its effects on the proposed method is much slighter than that of *δ*, thus, we select *ξ* = 0.4 for both simulated and experimental fMRI data (*δ* = 0.4).

Note we have retuned the parameters for the four algorithms obtained by removing each of constraints from the proposed slcTKD, as shown in Fig. 12, which is based on the abovementioned parameter selection. We do not change the sparsity constraint parameters *δ* and *p* representing the spatial sparsity levels of the simulated (*δ* = 2.5) and experimental (*δ* = 0.4) data (both: *p* = 0.3), and use the same ADMM and HQS parameters (*η* = 1.3 and *ξ* = 0.4) as used by slcTKD. For the sparsity constraint parameters *λ* and *γ*, previous choices (*γ* = 0.6, *λ* = 0.4) also achieve better performance. Finally, we change to select *μ*_*S*_ = 0.1 for slcTKD-||S||_F_, and *μ*_*B*_ = 0.1 for slcTKD-||B||_F_ to achieve higher $\left|{\bar{\rho }}_{c}\right|$ values for both shared SMs and TCs from both simulated and experimental data.

### C. Computational Complexity and Convergence

Based on the proposed slcTKD model in (2) and its algorithm implementation in Table II, we derive the total time and space complexity per iteration. They are *O*(*K*(*N*^3^ + *TN*^2^ + *VN* + *VT* + *VN*^2^ + *VTN*)) and *O*(*N*^2^ + *TN* + *KN*^2^ + *VN* + *KVT*) (sorted from smallest to largest), respectively. Table IV shows details for the two factor matrices and the core and residual tensors. The spatial sparsity constraint ||**S**||_*p*_ causes *VN* additional time and space complexity per iteration when estimating the shared SMs, resulting in slight increases of time complexity (approximately 1/*T*; simulated data: 1%; experimental data: 0.6%) and space complexity (approximately *N/KT*; simulated data: 2%; experimental data: 3%).

**Fig. 15.**
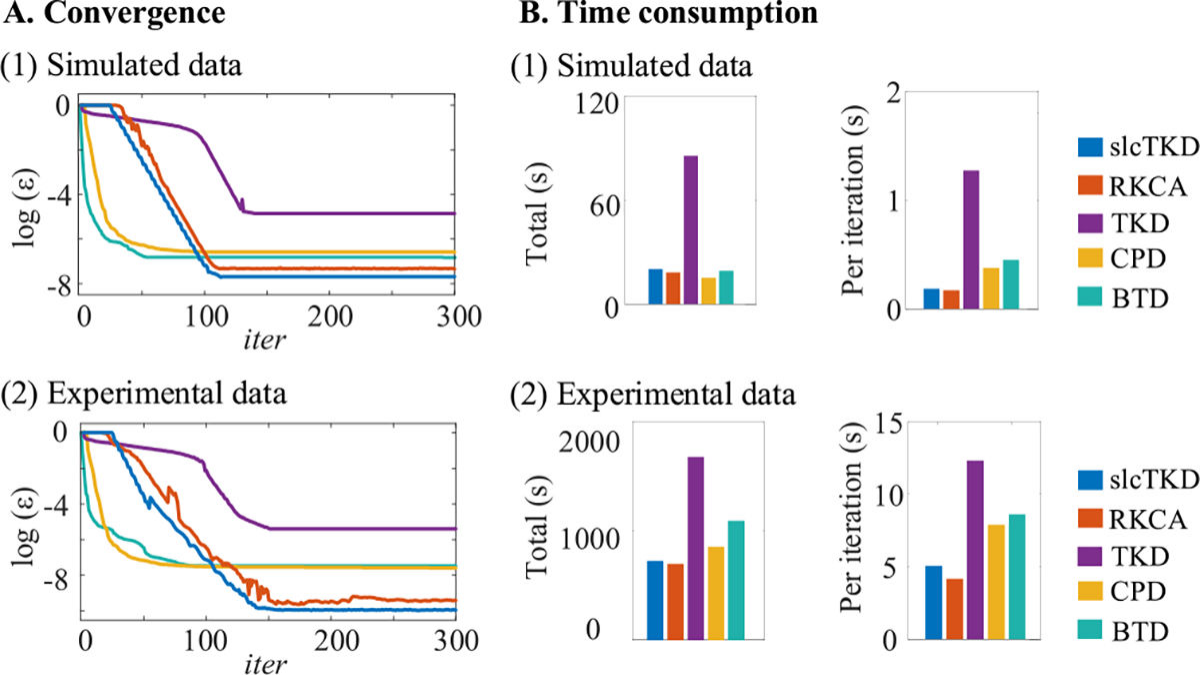
Convergence (A) and time consumption (B) of slcTKD, RKCA, TKD, CPD, and BTD for simulated data (1) and experimental data (2).

We carry out experiments on Intel 6226R CPU, 256G RAM, windows 10 (64-bit) using MATLAB 2020. Fig. 15 shows the convergence (*iter* = 1 ~ 300) of each algorithm in terms of the residual *ε* and the time consumption (total and per iteration) for both simulated and experimental fMRI data. We normalize *ε* to the range of 0 to 1 and show its logarithm value to have a clear visualization. CPD and BTD show faster convergence, followed by the proposed slcTKD, RKCA and TKD. However, slcTKD and RKCA consume similarly smaller running time than TKD, BTD and CPD for the experimental fMRI data, due to smaller time consumption per iteration. The proposed slcTKD algorithm yields the smallest residual among the five algorithms by incorporating the spatial sparsity constraint conforming to the intrinsic characteristic of fMRI data.

### D. Future Work

Our future work includes the following. First, we will further improve the Tucker-2 model to represent the multi-subject fMRI data and to extract more information such as individual time delays. Second, we will extend the proposed method to complex-valued fMRI data since the fMRI data is actually complex-valued and recent researches including ours [26] decompose complex-valued fMRI data, yielding promising results [26], [49]. Third, an adaptive sparsity constraint parameter selection method will be explored to discover the links between sparsity parameters and the experimental fMRI data. Finally, we will apply the proposed method to resting-state fMRI data or fMRI data based on regions of interest (ROIs) to analyze patients with neurological disorders.

## Figures and Tables

**TABLE I:** Definitions of Notations

Notation	Meaning
×_n_	mode-n product
❬·❭	inner product of matrices
|·|	element-wise absolute value
*sign*(·)	element-wise sign function
◦	Hadamard product
./	element-wise division
†	pseudo-inverse of matrix

**TABLE II:** Implementation of the Proposed slcTKD Algorithm

**Input:** multi-subject fMRI data $\underline{X}\in R^{V\times T\times K}$; the model order *N*; the maximum number of iteration *iter*_max_; the iteration number of Newton-Raphson method *iter_y*; termination thresholds of the error *ε*_min_ and the relative error change Δ*ε*_min_; parameters *δ*, *p*, *γ*, *λ*, *η*, and *ξ*
Initialize factor matrices **S** and **B**, core tensor **G**, multipliers **U**, **W**, and **Q**, penalty parameters *α*, *β*; **Y** = **S**, **R** = **G**, calculate **E** using $\underline{X}-\underline{G}\times_{1}S\times_{2}B$ based on (1); *ε*^0^ = 1; Δ*ε*^0^ =1; *iter*=0;
**While** *iter < iter*_max_, *ε^iter^* > *ε*_min_, or Δ*ε^iter^* > Δ*ε*_min_ do
*iter=iter*+1;
Update **B** using (6);
Update **S** using (8);
for *iter*_1_=1: *iter_y*
Calculate **Y** using (11);
end
Update **G** using (12);
Update **R** from discrete-time Sylvester equation in (13);
Update **E** using (14);
Update multipliers and parameters:
Update **U**, **W**, and **Q** using (15), (16), and (17);
Update *α* and *β* using (18) and (19);
$\varepsilon^{iter}=\|\underline{H}-\underline{G}\times_{1}S\times_{2}B-{E\|}_{F}/{\|\underline{X}\|}_{F}$;
Δ*ε^iter^* =|*ε*^*iter*−1^ − *ε^iter^* |/*ε*^*iter*−1^;
**End** while
Output: **S**, **B**, **G**;

**TABLE III:** A Comparison of the Proposed slcTKD, RKCA, TKD, CPD, and BTD in Terms of the Total Number of Activated Voxels and the Voxel Number Inside the Spatial Reference for The Two Shared SM Estimates Displayed In Fig. 6. The Maximum Values Are Shown in Bold

		slcTKD	RKCA	TKD	CPD	BTD

Task	Total voxels	3376	**5266**	4970	1856	3549
	Inside reference	**2045**	1353	1560	868	1509

DMN	Total voxels	3607	4548	**5124**	2952	4563
	Inside reference	**2534**	2011	1692	1727	2317

**TABLE IV:** Time and Space Complexity of Matrices and Tensors per Iteration of the Proposed Method. Those Caused by ||**S**||_*p*_ Are Shown in Bold

	Time complexity	Space complexity
**S**	*O(K(N^3^+TN^2^+**VN**+VT+VTN))*	*O(TN+KN^2^+**VN**+VN+KVT)*
**B**	*O(K(N^3^+TN^2^+VT+VTN))*	*O(TN+KN^2^+VN+KVT)*
**G**	*O(KN^2^)*	*O(N^2^+KN^2^)*
**E**	*O(K(VT+VN^2^+VTN))*	*O(TN+KN^2^+VN+KVT)*
